# A 5-km-thick reservoir with > 380,000 km^3^ of magma within the ancient Earth's crust

**DOI:** 10.1038/s41598-022-19915-w

**Published:** 2022-09-19

**Authors:** Rais Latypov, Sofya Chistyakova, Richard A. Hornsey, Gelu Costin, Mauritz van der Merwe

**Affiliations:** 1 School of Geosciences, University of the Witwatersrand, Johannesburg, South Africa; 2 Richard Hornsey Consulting (Pty) Ltd, Paternoster, South Africa; 3 Department of Earth Science, Rice University, Houston, TX USA; 4 Port Albert, South Africa

**Keywords:** Planetary science, Solid Earth sciences

## Abstract

Several recent studies have argued that large, long-lived and molten magma chambers may not occur in the shallow Earth’s crust. Here we present, however, field-based observations from the Bushveld Complex that provide evidence to the contrary. In the eastern part of the complex, the magmatic layering continuously drapes across a ~ 4-km-high sloping step in the chamber floor. Such deposition of magmatic layering implies that the resident melt column was thicker than the stepped relief of the chamber floor. Prolonged internal differentiation within this thick magma column is further supported by evolutionary trends in crystallization sequence and mineral compositions through the sequence. The resident melt column in the Bushveld chamber during this period is estimated at > 5-km in thickness and > 380,000 km^3^ in volume. This volume of magma is three orders of magnitude larger than any known super-eruption in the Earth’s history and is only comparable to the extrusive volumes of some of Earth’s large igneous provinces. This suggests that super-large, entirely molten, and long-lived magma chambers occur, at least occasionally, in the geological history of our planet. Therefore, the classical view of magma chambers as ‘big magma tanks’ remains a viable research concept for some of Earth’s magmatic provinces.

## Introduction

For over a century, the classic paradigm of volcanology and igneous petrology has been premised upon the existence of magma chambers, filled by crystal-free melt, forming ‘big tanks’^1–10^. Such magma chambers gradually lose heat and crystallize from all margins inwards and occasionally supply overlying extrusive centres (volcanoes or fissures) with magma that erupts onto the Earth’s surface^1–10^. This founding concept has, however, been recently challenged on the basis of observations and evidence from various disciplines. The most often-cited evidence is derived from geophysical surveys that are unable to conclusively identify any present-day magma chambers with large volumes of eruptible melt^11^. This is supported by thermal modelling^12^, which indicates that the formation of a large magma body within the upper crust is physically problematic, because it requires a magma accumulation rate that is 1–2 orders of magnitude greater than determined through geochronology^11^. In addition, out-of-sequence geochronology has been used to argue that the known mafic–ultramafic plutons do not require the existence of large magma chambers but could be produced as a stack of randomly-emplaced amalgamated sills^13–15^. The conclusion from these studies is that large, predominantly molten magma chambers are likely either transient^16^ or non-existent^11,12^ in the geological history of the Earth. As an alternative, it is proposed that intracrustal melt is stored within intergranular pockets of crystal-rich mushes that occupy almost the entire crust, from the Moho towards surface^16–21^. Periodic tectonic destabilization of the mush may produce small, discrete melt lenses that subsequently aggregate, ascend and erupt as lava. There are, however, some observations from magmatic complexes^22–24^ as well as thermal modelling constraints^12^ that conflict with this emerging paradigm^16–21^. Here we present one well-constrained example from the Bushveld Complex, indicating that the magma chamber appears to have contained, during one stage of its evolution, an enormous volume of resident melt that slowly crystallized from the base upwards to produce a continuous sequence of chemically stratified cumulate rocks.

### Incremental growth of the Bushveld Complex

**Figure 1:**
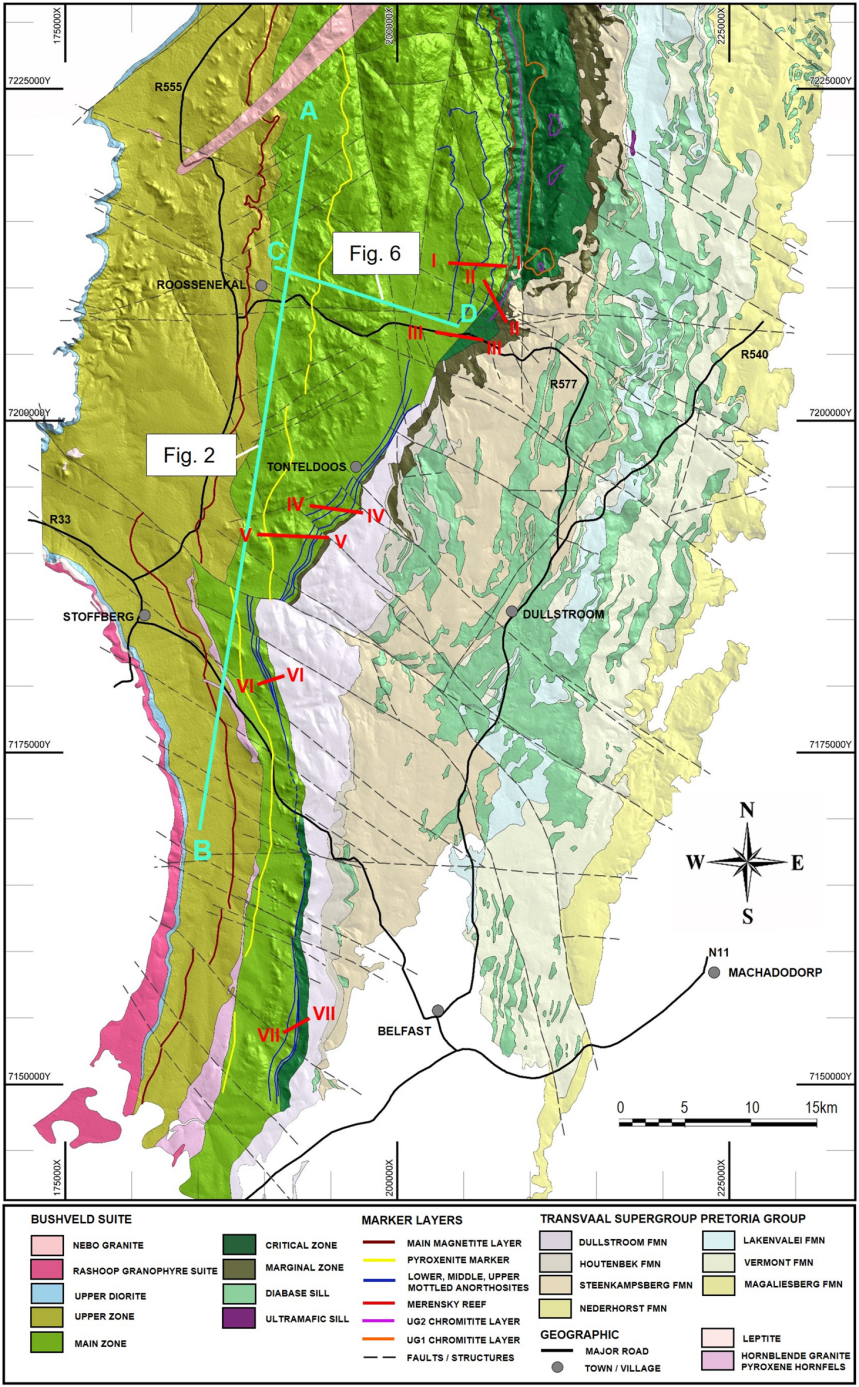
The geological map of the southeastern part of the Bushveld Complex in the Tonteldoos area. The complex transgresses upwards through the Transvaal Supergroup over the northern 35 km of this sector and the floor of the complex steps up by approximately 6 km. The basal part of the Main Zone has three continuous markers layers termed the Lower, Middle, and Upper Mottled Anorthosites that extend along the entire area. The position of cross-sections in Figs. 2 and 6 and seven major traverses across Anorthosite Markers in Figs. 3, 4, 5 (see also Supplementary Data) are indicated. Regional geology digitised from the 1:250,000 scale geology maps 2528 Pretoria and 2530 Barberton (Geological Survey of South Africa), with additional detail from mapping by Van der Merwe^49^, Bevington and Hornsey (2010, Nuplats Ltd, unpublished mapping), and Latypov and Chistyakova (2021, unpublished mapping). The figure was prepared by Richard Hornsey using Micromine 2021 Release 21.5.

The 2.05 billion-year-old Bushveld Complex in South Africa is the largest mafic–ultramafic layered intrusion into the Earth’s crust. It occupies an area that most likely exceeds 100,000 km^2^ and extends ~ 450 km east–west and ~ 350 km north-south^25–28^. Despite its enormous size, this complex is merely the remaining portion of an originally much larger intrusion that has subsequently been eroded to an unknown extent by surface processes. The complex consists of several parts, of which the western, eastern and northern limbs are the largest, and is stratigraphically subdivided into five major units—the Marginal, Lower, Critical, Main, and Upper Zones, comprising a total thickness of about 7 to 9 km^25^. The Bushveld Complex is widely considered to be a typical example of an open-system magma chamber^29^. Apart from the marginal rocks, its four principal zones are attributed to major replenishing events, with numerous smaller magma recharges contributing to the formation of these zones. During this process, the magma chamber incrementally increased in size through both vertical and lateral inflation^29–31^. All major replenishing events are marked by regionally extensive magmatic disconformities, local erosive unconformities into previous strata, significant isotopic shifts, and notable changes in whole-rock and mineral compositions^29,32–34^. These relationships are best exemplified by the Main Zone (MZ) at the Tonteldoos area of the southeastern Bushveld Complex (Fig. 1), which has a much larger lateral extent than the underlying Critical Zone (CZ), as indicated by the MZ’s direct onlapping of the floor rocks in many places. The MZ is commonly attributed to a large influx of new magma that significantly expanded the chamber in both vertical and lateral extent, producing a regional disconformity^29^ and locally prominent unconformities with pre-existing CZ cumulates^32,35^. The base of the MZ is also marked by a substantial isotopic shift towards more radiogenic whole-rock ^87^Sr/^86^Sr ratios^36^. The laterally extensive Pyroxenite Marker in the uppermost part of the MZ indicates another major expansion of the chamber through a magma replenishment and mixing event that caused an isotopic shift towards less radiogenic whole-rock ^87^Sr/^86^Sr ratios^37,38^ and an increase in both the An-content of plagioclase and Mg-number of pyroxenes^39–42^. Successive crystallisation of the entire Bushveld Complex from the Lower and Upper Critical Zone to the Main Zone occurred within ca. 1 million years, between 2055.91 ± 0.26 Ma and 2054.89 ± 0.37 Ma^43^. Attempts to place stricter constraints on the crystallisation of the complex using high-precision U–Pb TIMS ages^13,15^ have proved problematic because zircon isotopic ages in these studies appear to be at odds with basic field relationships^44^.

### The resident melt column of the Bushveld Complex

**Figure 2:**
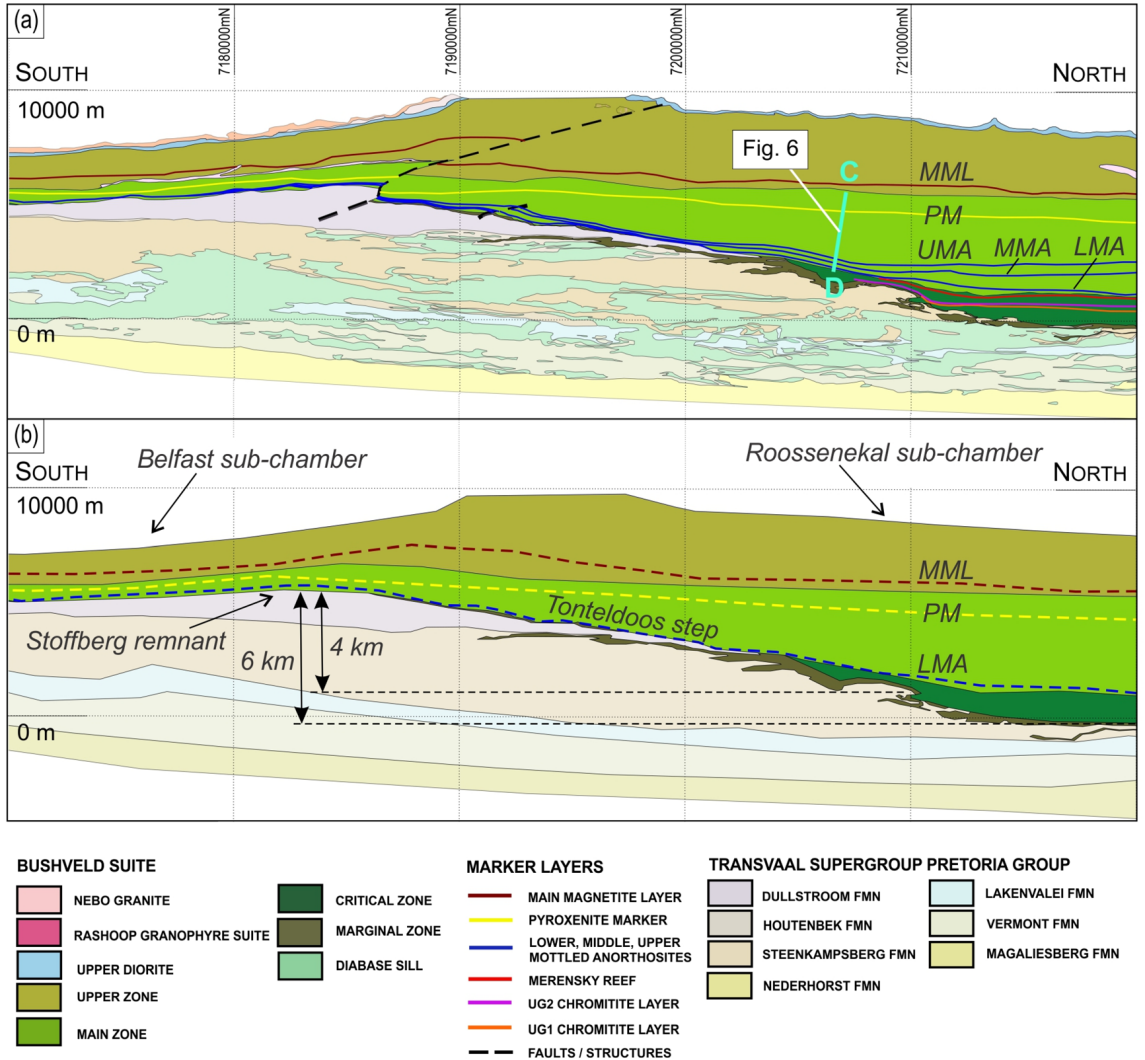
Geological along-strike section of the southeastern part of the Bushveld Complex in the Tonteldoos area. (**a**) The section is of the transect line AB in Fig. 1, looking at − 11° towards 270° azimuth. The section was constructed by rotating the 3D model to view the detailed geology of the complex and its immediate footwall in their true orientation. The section is not vertically or horizontally exaggerated and shows the true lithology morphology. (**b**) The schematic section prior to faulting highlights several important features of the complex: (1) the existence of the Roossenekal and Belfast sub-chambers with the intervening Stoffberg remnant of non-deformed host rocks, (2) the ~ 4-km and ~ 6-km thick vertical distance between the summit of the Stoffberg remnant with the Lower Mottled Anorthosite and the floor of the Roossenekal sub-chamber, respectively, (3) the concave geometry of the Pyroxenite Marker indicating that instantaneous top of the cumulate pile in the two sub-chambers was gently basinal with regards to the Stoffberg remnant. LMA, Lower Mottled Anorthosite; MMA, Middle Mottled Anorthosite; UMA, Upper Mottled Anorthosite; PM, Pyroxenite Marker; MML, Main Magnetite Layer. The CD line in (**a**) shows the location of the cross-section in Fig. 6. The figure was prepared by Richard Hornsey using Micromine 2021 Release 21.5.

The stratigraphy of the Bushveld Complex is most commonly thought to have progressively accumulated from the overlying resident melt by deposition of crystals on the chamber floor^6,29,45^ although there are several alternative views^13,15,46^. A critical unknown parameter is the volume of resident melt at any particular time during the evolution of the magma chamber^26,47^. We present here a potential solution through field mapping of the southeastern Bushveld Complex in the Tonteldoos area (Fig. 2), where the resident melt column thickness at the time of MZ crystallization may be assessed. The field mapping and 1:250,000 scale regional geology maps of the study area are compiled into a 3D model that includes and respects all mapped relationships between various geological units. The along-strike section in Fig. 2 is viewed down the dip of the stratigraphy and thereby preserves all the relationships between the intrusion and its host rocks, without loss of any details. It reveals that the host stratigraphy along this section dips at 11° to the west. The sedimentary host rock sequence was intruded by precursor Bushveld Complex sills that are sharply truncated by the main plutonic phase of the Bushveld Complex. The mapped geology exposes no evidence for any major syn-magmatic (e.g., the floor subsidence) or post-emplacement (e.g., fault displacement) structural deformation of the country rocks (Fig. 2), implying that the section was emplaced as shown and preserves all primary igneous field relationships.

Two notable features illustrated by Fig. 2 provide crucial constraints. The first relates to the geometry of the chamber that has been previously subdivided and described as two large basinal structures^39^. We refer to them, however, as the Roossenekal and Belfast sub-chambers because they lack any associated structural deformation or down-warping of their sedimentary host rocks (Fig. 2). The floors to both sub-chambers are concave upwards and juxtapose an intervening Stoffberg remnant of non-deformed host rocks. The floor contact of the Roossenekal sub-chamber has an impressive ~ 6-km-high shelf relief (termed the Tonteldoos step; Fig. 2b) with a ~ 10° slope across ~ 35 km up to the summit of the Stoffberg remnant. This topographic relief of the floor contact constrains a sub-chamber that was emplaced along an angular discordance to its host rock stratigraphy^48^. From north to south, the floor of the Roossenekal sub-chamber initially overlies the Steenkampsberg Formation, then transgresses the Houtenbek Formation and finally onlaps the Dullstroom Formation. The Roossenekal sub-chamber thus attains its maximum thickness in the north and thins towards the south where the CZ eventually terminates against the intrusion floor. The MZ and UZ extend across the entire extent of the two sub-chambers. The MZ in the Roossenekal sub-chamber directly onlaps onto the sedimentary and volcanic floor rocks (Fig. 2).

**Figure 3:**
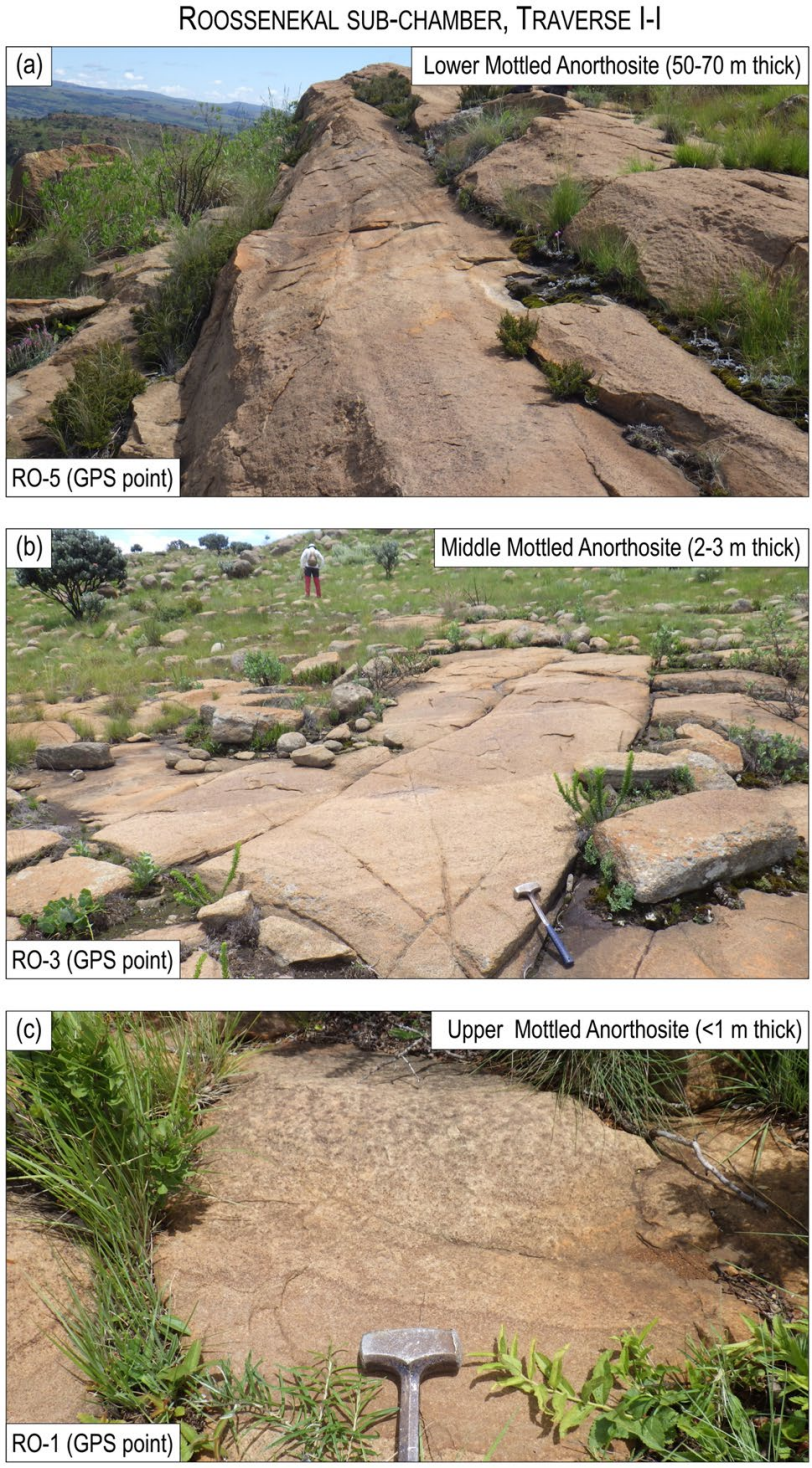
Photos of field outcrops of the three Anorthosite Markers along the traverse I-I of the Roossenekal sub-chamber of the southeastern part of the Bushveld Complex. All photos were taken by Sofya Chistyakova or Rais Latypov.

**Figure 4:**
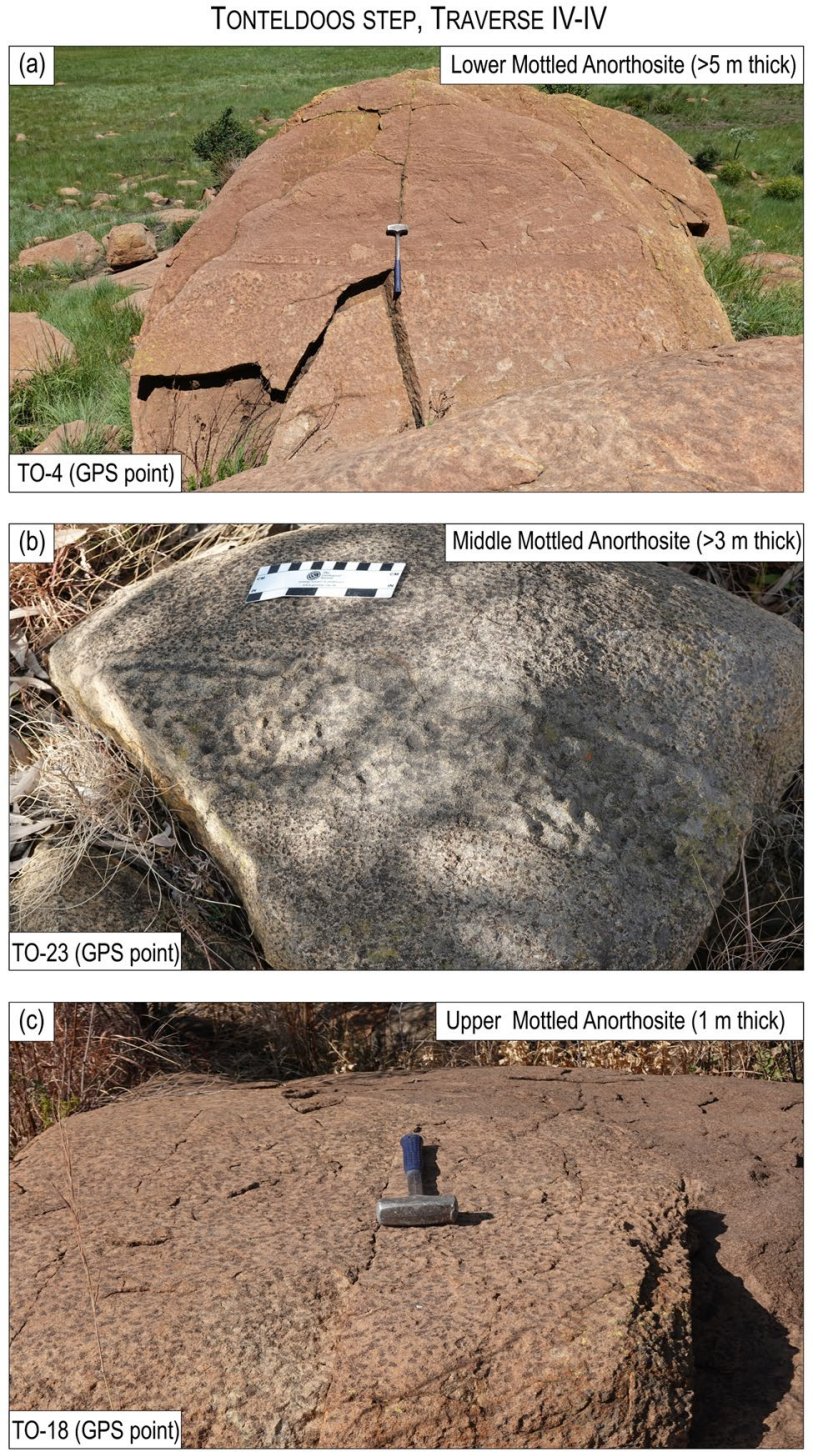
Photos of field outcrops of the three Anorthosite Markers along the traverse IV-IV at the Tonteldoos step of the Roossenekal sub-chamber of the southeastern part of the Bushveld Complex. All photos were taken by Sofya Chistyakova.

**Figure 5:**
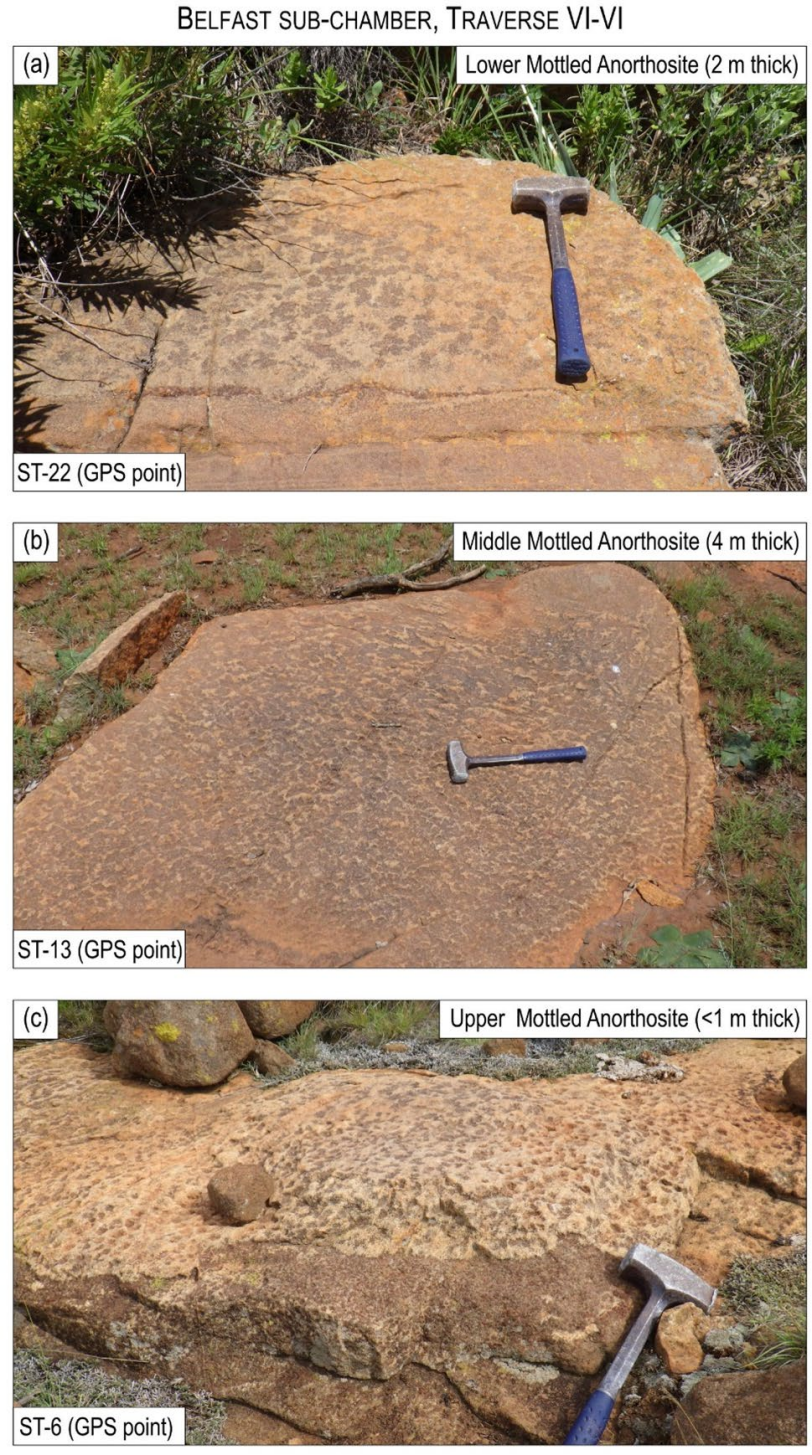
Photos of field outcrops of the three Anorthosite Markers along the traverse VI-VI of the Belfast sub-chamber of the southeastern part of the Bushveld Complex. All photos were taken by Sofya Chistyakova.

The second feature relates to the igneous layering, defined by prominent layers termed the Lower, Middle and Upper Mottled Anorthosites (LMA, MMA and UMA), which occur close to the base of the MZ^49^. Although it is widely assumed that the MZ layering abuts the floor contact of the country rocks^37,39,40^, the Anorthosite Markers do not terminate but drape across the entire Roossenekal and Belfast sub-chambers, including the Stoffberg remnant^49^. The Anorthosite Markers have been mapped by the authors along strike at seven field traverses through the basal part of the MZ in both sub-chambers (Figs. 1, 3, 4, 5; Extended Data Fig. 1). Field mapping indicates that from north to south the Anorthosite Markers tend to decrease in thickness, without any systematic changes in their textures. Importantly, the presence of the Anorthosite Markers across the entire area indicates that the deposition of MZ cumulates occurred synchronously across the deepest (i.e., Roossenekal sub-chamber base) and shallowest (i.e., Stoffberg remnant summit) parts of the sub-chamber (Fig. 2). This occurred despite the elevation difference between the two contrasting depositional places being ~ 4 km (Fig. 2b). This relationship cannot be due to syn- or post-emplacement subsidence of the Roossenekal sub-chamber and its host stratigraphy because the local and regional geology shows no evidence for the depression of the floor rocks, either through faulting or magma emplacement (Figs. 1 and 2).

**Figure 6:**
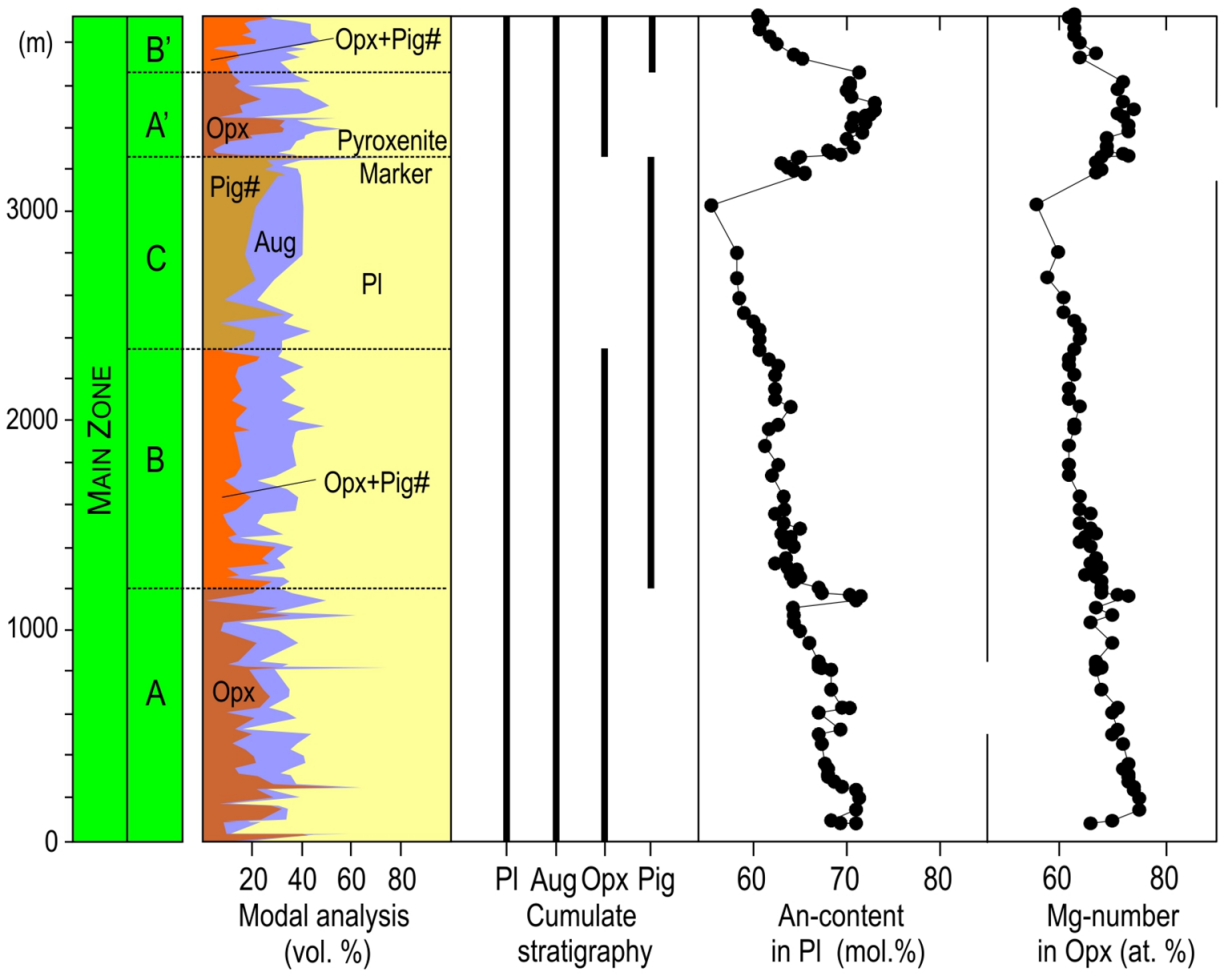
Modal abundances, cumulate stratigraphy and compositional variations of minerals through the Main Zone in the Tonteldoos area of the southeastern part of the Bushveld Complex. The section is approximately along the transect line CD in Figs. 1 and 2. The lower stratigraphy is characterized by a systematic crystallization sequence Opx + Aug + Pl (A), Opx + Aug + Pig + Pl (B), and Aug + Pig + Pl (C). The uppermost stratigraphy is marked by re-appearance of cumulus Opx at the Pyroxenite Marker so that the crystallization sequence Opx + Aug + Pl (A′) and Opx + Aug + Pig + Pl (B′) is repeated. The lower stratigraphy shows systematic evolutionary trends in composition of plagioclase (decrease in An-content, 100Ca/(Ca + Na)) and orthopyroxene (decrease in Mg-number, 100 Mg/(Mg + Fe)). The transition to the Pyroxenite Marker is characterized by a gradual compositional reversal towards higher An-content of plagioclase and Mg-number of orthopyroxene. Mg-number in subzone C is for orthopyroxene that is produced by inversion of primary pigeonite. All compositional data in (a–e) are from Gruenewaldt^41^ and are summarized in Supplementary Data Table 1. Pl, plagioclase; Opx, orthopyroxene; Aug, augite; Pig#, inverted pigeonite. The figure was prepared by Sofya Chistyakova using CorelDRAW (version 18.1.0.690).

Igneous layering in mafic intrusions results from deposition of crystals from the overlying resident melt due to gravity settling^4,50–52^ or in situ crystallization^9,53–56^ onto the chamber floor. This indicates that in order to blanket the topographic relief of a temporary floor of the Bushveld chamber with igneous layering (i.e., LMA), the resident melt column must have been thicker than the ~ 4.0 km height of the Tonteldoos step. The lithological interpretation is supported by a systematic decrease in An-content of plagioclase and Mg-number of orthopyroxene through the ~ 3.0-km-thick MZ stratigraphy of the Roossenekal sub-chamber, indicating internal differentiation of a resident melt column that was thicker than the crystallised sequence (Fig. 6). The transition to the overlying Pyroxenite Marker is defined by an up to 0.5-km-thick reversal towards more primitive mineral composition (Fig. 6), which has been interpreted to result from mixing of a residual MZ melt with new magma entering the chamber^29,40,42,57,58^, causing further vertical expansion. Mass balance calculations based on Sr-isotopic data indicate that the residual melt comprised 60–70% of the resulting hybrid magma^29,37^, which subsequently crystallized to form a > 3.0 km thick sequence overlying the Pyroxenite Marker (Fig. 2). If correct, the residual melt of the MZ must still have been ~ 2-km-thick prior to the Pyroxenite Marker magma influx, thereby indicating an initial ~ 5 km thickness of the MZ melt column; consistent to earlier estimates based upon thermal modelling of the Bushveld Complex^26^. We concur with previous studies^39,59^ that the instantaneous top of the cumulus pile during deposition in this region was gently basinal, with the Stoffberg remnant partially separating the Roossenekal and Belfast sub-chambers. This is best indicated by the concave geometry of the Pyroxenite Marker within the Roossenekal sub-chamber, resulting in this layer being almost 2 km stratigraphically lower at the centre of this sub-chamber, compared to the Stoffberg remnant (Fig. 2). This field evidence implies that the ~ 1.0 km and ~ 3.0 km thick MZ in the Roossenekal and Belfast sub-chambers, respectively, formed synchronously from the same interconnected resident magma, further substantiated by similar An-content of plagioclase at the base of the MZ at both sub-chambers (67.5%^39,60^ and 71%^40,41^, respectively). The greater thickness of MZ cumulates in the Roossenekal sub-chamber may be related to the greater thickness of this unit, due to redeposition of crystals inside this depression and/or prevailing crystallization in the deeper parts of the magma chamber, due to pressure-induced increase in the liquidus temperature of the melt^9,54,61^.

**Figure 7:**
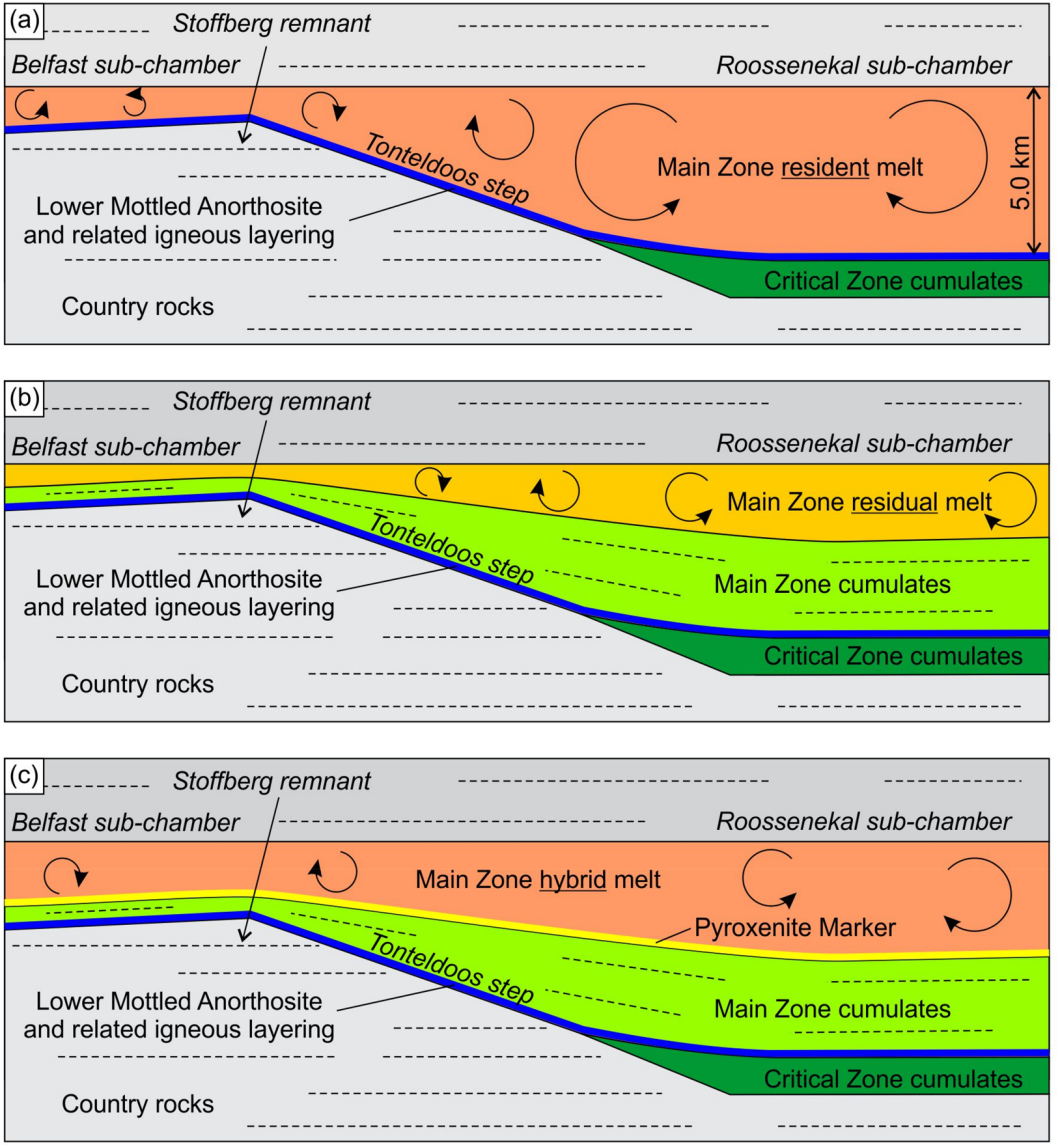
Model for the proposed crystallization history of the Main Zone in the Tonteldoos area of the southeastern part of the Bushveld Complex. (**a**) At onset of crystallization, the MZ resident melt column had a total thickness of about 5 km. This resulted in simultaneous deposition of the Lower Mottled Anorthosite along the entire extent of the Roossenekal and Belfast sub-chambers, including the Tonteldoos step and Stoffberg remnant. (**b**) Internal crystallization and differentiation of the MZ resident melt from the base upwards produced a continuous sequence of chemically-stratified cumulate rocks. The instantaneous top of the cumulate pile in the chamber was gently basinal with the Stoffberg remnant separating two sub-chambers. (**c**) Mixing of the MZ residual melt with a new magma influx resulted in the formation of the MZ hybrid melt that produced the laterally extensive Pyroxenite Marker with an associated reversal in mineral compositions. The total volume of resident melt before onset of crystallization is estimated at > 380,000 km^3^ which allows to classify the Bushveld chamber as a ‘big tank’ open-system within the Earth’s crust. The figure is not to scale and is for illustrative purposes. The figure was prepared by Rais Latypov using CorelDRAW (version 18.1.0.690).

Rather than implying that the entire ~ 5.0 km thick column formed from a single large magma influx, it is proposed that the intrusion progressively grew to its final size (from the Roossenekal sub-chamber towards the Belfast sub-chamber) through emplacement of numerous magma influxes, yet over a much shorter time scale than solidification. During this period of repeated injections, each replenishment effectively mixed with the resident melt in the chamber, thereby delaying or impeding the onset of crystallization. Thus, crystallization commenced within a completely filled, large and homogenized magma chamber, which can thereby be modelled as having crystallized as a ‘single pulse’ of magma (Fig. 7a). Our model is therefore substantially different from those where the melt crystallization and cumulate pile growth of the MZ occurred concurrent to magma chamber replenishment^37^. Our model conforms better to liquidus phase equilibria predictions for a basaltic parent, such as systematic changes in pyroxene assemblages (e.g., Opx-Aug through Opx-Aug-Pig to Aug-Pig)^62^ and continuous decreases in both the An-content and Mg-number of cumulus plagioclase and orthopyroxene, respectively (Figs. 6 and 7b). Both the crystallization sequence and mineral compositional trends are reproduced by fractional crystallization of the parental melt (Extended Data, Extended Data Fig. 2) using the alphaMELTS software. It should be noted, however, that the modelling results are not unique (e.g., they are greatly dependent on the choice of a parental melt composition) and cannot, therefore, be considered as solid evidence of the model developed in this study. Such unidirectional evolutionary trends are also inconsistent with models that attribute the formation of the MZ to externally-derived crystal-rich slurries, because these would result in either constant^63^ or irregular^64^ trends in mineral compositions through the MZ stratigraphy. It is quite conceivable, however, that during protracted fractionation of the MZ resident melt, the chamber may have been further replenished by additional minor magma pulses with or without phenocrysts since minor local reversals in the An-content of plagioclase are discernable within the overall decreasing trend (Fig. 6). In fact, the Anorthosite Markers themselves may be associated with magma chamber replenishments^65^. Such occasional and relatively small magma chamber recharges do not, however, modify our major conclusion with respect to the initial resident melt column thickness, prior to the onset of crystallization at the first anorthosite marker (i.e., LMA in Fig. 2). It was only after a protracted and relatively quiet period of continuous crystallization of the MZ, that a major influx of orthopyroxene-saturated magma incrementally mixed with the magma chamber’s resident melt, while concomitantly crystallizing a succession of cumulates both below and above the Pyroxenite Marker (see Thermodynamic modeling in Extended Data).

### Implications for the ‘big tank’ magma chamber paradigm

The 5-km-thick resident melt column that produced the chemically stratified MZ cumulate sequence, including its three prominent Anorthosite Markers, indicates that during this stage the Bushveld chamber was exceptionally large and entirely molten (Fig. 7). The formation of such a cumulate sequence, at a typical solidification rate for large mafic intrusions (~ 1 cm/year^50,66^) would take ~ 300,000 years indicating that the intrusion was also long-lived. These conclusions are at odds with an emerging paradigm that such ‘big tank’ magma chambers were ephemeral at any given time throughout Earth’s geological history^16,19,20,67,68^. The total volume of resident MZ melt may be estimated as follows. The MZ melt column varied between ~ 5.0 km in the thicker and, at least, 1.0 km thick in the thinner parts of the intrusion. Based on reconstructions of the lateral extent of the MZ^29^, it is estimated that the thicker areas occupied ~ 70% of the MZ, and the remaining ~ 30% were thinner zones. The estimated Bushveld Complex area is approximately 100,000 km^3,26,28^ and therefore the total volume of the MZ resident melt is ~ 380,000 km^3^ (5 km * 70,000 km^2^ + 1.0 km * 30,000 km^2^). This volume is several orders of magnitude larger than the largest ignimbrite/tuff super-eruptions in Earth’s history (e.g., Bishop tuff—600 km^3^ and Youngest Toba eruption—up to 13,200 km^3^)^69^. It is only comparable to estimates of some of Earth’s large igneous provinces, such as the Karoo (367,000 km^3^)^70^ and Afar (350,000 km^3^)^71^. Thus, during emplacement of the MZ, the Bushveld magma chamber was a repository of an enormous volume of resident melt and may be regarded as a ‘big tank’ open-system within the Earth’s crust. Therefore, the current tendency in modern volcanology^16,19,20^ and petrology^13–15^ to discard the existence of such large and molten magma chambers^4–7^ appears to be premature. There is also no compelling reason to believe that ‘big tank’ magma chambers, such as the 2.05 Ga Bushveld Complex, are restricted to a long-forgotten past of our planet, such as the Precambrian. This is indicated by the 55 Ma Skaergaard intrusion in Greenland whose spectacular chemical stratigraphy indicates that before the onset of crystallization it was a ‘big tank’ of crystal-free tholeiitic parent magma up to 4 km in thickness and up to 300 km^3^ in volume^72,73^. Another example is the 1.3 Ga Kiglapait intrusion in Labrador with 3500 km^3^ of magma in a > 8 km thick magma chamber that shows a continuous differentiation sequence with little or no magma recharge^74,75^. It is therefore conceivable that such magma chambers have developed throughout the entire Earth’s evolution. Even if some regions of the Earth’s crust may behave as giant crystal mushes (e.g., mid-ocean ridges or deep roots of continental arcs)^16,17,20,76^, this does not automatically imply that ‘big tank’ magma chambers are absent from other regions (e.g., stable cratons with layered intrusions)^5–7^. Moreover, since layered intrusions such as the Bushveld Complex are rare throughout geological time^77^, it is not surprising that there are currently no active examples of large and molten magma chambers in Earth’s crust which can be detected geophysically^11^.

## Methods

### Map and cross-sections constructions

The maps were created by digitally capturing the 1:250,000 geological survey maps (2528 Pretoria, and 2530 Barberton, Geological Survey of South Africa) into Micromine software (http://www.micromine.com). Additional details come from mapping by Van der Merwe^49^, Bevington and Hornsey (2010, Nuplats Ltd, unpublished mapping), and Latypov and Chistyakova (2021, unpublished mapping). Micromine is a commercially available geological software package widely used for exploration and mine planning to model geological datasets in 3d space. The version used for the latest iteration of the model was Micromine 2021 Release 21.5. The maps were digitally captured as polygons, which were then draped onto a digital terrain model downloaded from the USGS Earth Explorer website (https://earthexplorer.usgs.gov). The terrain data were used to create a digital terrain model wireframe (DTM) that was cut into separate entities using the geological polygons and attributed according to the lithological unit. To create the geological section, the geological model was rotated using Micromine to enable visualisation of the data as a section looking down the plunge of the Bushveld Complex. The detailed geology was then manually digitised from the 3-dimensional section as a planar section that was then attributed to show the lithological units using the same colour scheme as for the plan. The imagery was exported from Micromine as formatted plans and sections. These were then imported into MSPowerpoint software, within which the labelling and legends were added. The final product was then exported as an image for inclusion into the research paper. The geological section therefore illustrates the entire stratigraphy and relationships between the Bushveld Complex and its host stratigraphy showing the “as-mapped” relationships and geometries in their correct spatial location. The maps are all in WGS84 Datum, UTM zone 36 South.

## Supplementary Information

Supplementary Information 1.

Supplementary Information 2.

Supplementary Information 3.

Supplementary Information 4.

Supplementary Information 5.

Supplementary Information 6.

Supplementary Information 7.

Supplementary Information 8.

Supplementary Information 9.

Supplementary Information 10.

Supplementary Information 11.

Supplementary Information 12.

Supplementary Information 13.

Supplementary Information 14.

Supplementary Information 15.

Supplementary Information 16.

Supplementary Information 17.

Supplementary Information 18.

Supplementary Information 19.

Supplementary Information 20.

Supplementary Information 21.

Supplementary Information 22.

## Data Availability

The authors declare that all relevant data are available within the article and its Supplementary Information Files.

## References

[1] Daly RA (1911). The nature of volcanic action. Proc. Am. Acad. Arts Sci..

[2] Bowen NL (1928). The Evolution of the Igneous Rocks.

[3] Wager LR, Deer WA (1939). Geological investigations in East Greenland, Part III: The petrology of the Skaergaard Intrusion, Kangerdlugssuaq, East Greenland. Med. Greenland.

[4] Wager LR, Brown GM (1968). Layered Igneous Rocks.

[5] Parsons I (1987). Origins of Igneous Layering.

[6] Cawthorn RG (1996). Layered Intrusions.

[7] Charlier B, Namur O, Latypov R, Tegner C (2015). Layered Intrusions.

[8] Marsh BD (1996). Solidification fronts and magmatic evolution. Mineral. Mag..

[9] Campbell, I. H. Fluid dynamic processes in basaltic magma chambers. In *Developments in Petrology* Vol. 15, 45–76 (Elsevier, 1996).

[10] Gudmundsson A (2012). Magma chambers: Formation, local stresses, excess pressures, and compartments. J. Volcanol. Geotherm. Res..

[11] Lundstrom CC, Glazner AF (2016). Silicic magmatism and the volcanic–plutonic connection. Elements.

[12] Glazner AF (2021). Thermal constraints on the longevity, depth, and vertical extent of magmatic systems. Geochem. Geophys. Geosyst..

[13] Mungall JE, Kamo SL, McQuade S (2016). U-Pb geochronology documents out-of-sequence emplacement of ultramafic layers in the Bushveld Igneous Complex of South Africa. Nat. Commun..

[14] Wall CJ (2018). The Stillwater Complex: Integrating zircon geochronological and geochemical constraints on the age, emplacement history and crystallization of a large, open-system layered intrusion. J. Petrol..

[15] Scoates JS (2021). Dating the Bushveld Complex: Timing of crystallization, duration of magmatism, and cooling of the world’s largest layered intrusion and related rocks. J. Petrol..

[16] Cashman KV, Sparks RSJ, Blundy JD (2017). Vertically extensive and unstable magmatic systems: A unified view of igneous processes. Science.

[17] Bachmann O, Huber C (2019). The inner workings of crustal distillation columns; the physical mechanisms and rates controlling phase separation in silicic magma reservoirs. J. Petrol..

[18] Cooper KM (2017). What does a magma reservoir look like? The “crystal’s-eye” view. Elements.

[19] Jackson MD, Blundy J, Sparks RSJ (2018). Chemical differentiation, cold storage and remobilization of magma in the Earth’s crust. Nature.

[20] Sparks RSJ (2019). Formation and dynamics of magma reservoirs. Philos. Trans. R. Soc. A..

[21] Bachmann O, Bergantz G (2008). The magma reservoirs that feed supereruptions. Elements.

[22] Kruger W, Latypov R (2020). Fossilized solidification fronts in the Bushveld Complex argue for liquid-dominated magmatic systems. Nat. Commun..

[23] Latypov R (2022). Chromitite layers indicate the existence of large, long-lived, and entirely molten magma chambers. Sci. Rep..

[24] Kruger W, Latypov R (2021). Magmatic karst reveals dynamics of crystallization and differentiation in basaltic magma chambers. Sci. Rep..

[25] Cawthorn RG, Charlier B, Namur O, Latypov R, Tegner C (2015). The Bushveld Complex, South Africa. Layered Intrusions.

[26] Cawthorn RG, Walraven F (1998). Emplacement and crystallization time for the Bushveld Complex. J. Petrol..

[27] Naldrett AJ, Wilson A, Kinnaird J, Yudovskaya M, Chunnett G (2012). The origin of chromitites and related PGE mineralization in the Bushveld Complex: New mineralogical and petrological constraints. Mineral. Deposita.

[28] Finn CA, Bedrosian PA, Cole JC, Khoza TD, Webb SJ (2015). Mapping the 3D extent of the Northern Lobe of the Bushveld layered mafic intrusion from geophysical data. Precambrian Res..

[29] Kruger FJ (2005). Filling the Bushveld Complex magma chamber: Lateral expansion, roof and floor interaction, magmatic unconformities, and the formation of giant chromitite, PGE and Ti-V-magnetitite deposits. Mineral. Deposita.

[30] Willemse J (1959). The ‘floor’ of the Bushveld Igneous Complex and its relationships, with special reference to the Eastern Transvaal. S. Afr. J. Geol..

[31] Eales HV (2002). Caveats in defining the magmas parental to the mafic rocks of the Bushveld Complex, and the manner of their emplacement: Review and commentary. Mineral. Mag..

[32] Chistyakova SYu, Latypov RM, Kruger FJ, Zaccarini F (2021). Transgressive nature and chilled margins of the Upper Zone in the western Bushveld Complex, South Africa. Canad. Mineral..

[33] Hasch M, Latypov R (2021). Too large to be seen: Regional structures in Lower and Middle group chromitites of the Bushveld Complex, South Africa. Ore Geol. Rev..

[34] Latypov R, Chistyakova S, van der Merwe J, Westraat J (2019). A note on the erosive nature of potholes in the Bushveld Complex. S. Afr. J. Geol..

[35] Scoon RN, Mitchell AA (2004). Petrogenesis of wiscordant magnesian dunite pipes from the central sector of the Eastern Bushveld Complex with emphasis on the Winnaarshoek Pipe and disruption of the Merensky Reef. Econ. Geol..

[36] Kruger FJ, Marsh JS (1982). Significance of 87Sr/86Sr ratios in the Merensky cyclic unit of the Bushveld Complex. Nature.

[37] Sharpe MR (1985). Strontium isotope evidence for preserved density stratification in the main zone of the Bushveld Complex, South Africa. Nature.

[38] Kruger FJ (1994). The Sr-isotopic stratigraphy of the western Bushveld Complex. Trans. geol. Soc. S. Afr..

[39] Cawthorn RG, Lundgaard KL, Tegner C, Wilson JR (2016). Lateral variations in plagioclase compositions, Main Zone, Bushveld Complex, South Africa: Evidence for slow mixing of magmas in basinal structures. Mineral. Mag..

[40] Setera JB, VanTongeren JA (2018). Lateral variability in the Upper Main Zone, Bushveld Complex, owing to directional magma recharge and emplacement from north to south. J. Petrol..

[41] Von Gruenewaldt G (1973). The Main and Upper zones of the Bushveld Complex in the Roossenekal area, eastern Transvaal. Trans. Geol. Soc. S. Afr..

[42] Nex PAM, Cawthorn RG, Kinnaird JA (2002). Geochemical effects of magma addition: Compositional reversals and decoupling of trends in the Main Zone of the western Bushveld Complex. Mineral. Mag..

[43] Zeh A, Ovtcharova M, Wilson AH, Schaltegger U (2015). The Bushveld Complex was emplaced and cooled in less than one million years—Results of zirconology, and geotectonic implications. Earth Planet. Sci. Lett..

[44] Latypov RM, Chistyakova SYu (2022). Misinterpretation of zircon ages in layered intrusions. S. Afr. J. Geol..

[45] Maier WD, Barnes S-J, Groves DI (2013). The Bushveld Complex, South Africa: Formation of platinum–palladium, chrome- and vanadium-rich layers via hydrodynamic sorting of a mobilized cumulate slurry in a large, relatively slowly cooling, subsiding magma chamber. Mineral. Deposita.

[46] Robb SJ, Mungall JE (2020). Testing emplacement models for the Rustenburg Layered Suite of the Bushveld Complex with numerical heat flow models and plagioclase geospeedometry. Earth Planet. Sci. Lett..

[47] Cawthorn RG (2018). A non-horizontal floor during accumulation of the Bushveld Complex—Evidence and implications. Lithos.

[48] Button A (1976). Stratigraphy and relations of the bushveld floor in the Eastern Transvaal. S. Afr. J. Geol..

[49] Merwe MV (2007). The occurrence of the critical zone along the exposed southeastern sector of the eastern Bushveld Complex. S. Afr. J. Geol..

[50] Morse SA (1986). Convection in aid of adcumulus growth. J. Petrol..

[51] Irvine TN (1980). Magmatic density currents and cumulus processes. Am. J. Sci..

[52] Latypov RM, Chistyakova SYu (2020). Origin of non-cotectic cumulates: A novel approach. Geology.

[53] McBirney AR, Noyes RM (1979). Crystallization and layering of the Skaergaard intrusion. J. Petrol..

[54] Campbell IH (1978). Some problems with the cumulus theory. Lithos.

[55] Latypov R, Chistyakova S, Page A, Hornsey R (2015). Field evidence for the in situ crystallization of the Merensky Reef. J. Petrol..

[56] Latypov RM, Chistyakova SYu, Namur O, Barnes S (2020). Dynamics of evolving magma chambers: Textural and chemical evolution of cumulates at the arrival of new liquidus phases. Earth Sci. Rev..

[57] Cawthorn RG, Meyer PS, Kruger FJ (1991). Major addition of magma at the Pyroxenite Marker in the Western Bushveld Complex, South Africa. J. Petrol..

[58] Vantongeren JA, Mathez EA (2013). Incoming magma composition and style of recharge below the Pyroxenite Marker, Eastern Bushveld Complex, South Africa. J. Petrol..

[59] Cawthorn RG (2013). The residual or roof zone of the Bushveld Complex, South Africa. J. Petrol..

[60] Lundgaard KL, Tegner C, Cawthorn RG, Kruger FJ, Wilson JR (2006). Trapped intercumulus liquid in the Main Zone of the eastern Bushveld Complex, South Africa. Contrib. Mineral. Petrol..

[61] Jackson, E. D. *Primary textures and mineral associations in the ultramafic zone of the Stillwater complex, Montana*. *Professional Paper* 106 https://pubs.er.usgs.gov/publication/pp358 (1961) 10.3133/pp358.

[62] Latypov RM (2001). Graphical analysis of the orthopyroxene-pigeonite-augite-plagioclase equilibrium at liquidus temperatures and low pressure. Am. Mineral..

[63] Roelofse F, Ashwal LD (2012). The Lower Main Zone in the Northern Limb of the Bushveld Complex—a >1·3 km thick sequence of intruded and variably contaminated crystal mushes. J. Petrol..

[64] Yao Z, Mungall JE, Jenkins MC (2021). The Rustenburg Layered Suite formed as a stack of mush with transient magma chambers. Nat. Commun..

[65] Latypov R (2020). Monomineralic anorthosites in layered intrusions are indicators of the magma chamber replenishment by plagioclase-only-saturated melts. Sci. Rep..

[66] Morse SA (2011). The fractional latent heat of crystallizing magmas. Am. Mineral..

[67] Glazner AF, Coleman DS, Gray W, Taylor RZ (2004). Are plutons assembled over millions of years by amalgamation from small magma chambers?. GSA Today.

[68] Coleman DS, Gray W, Glazner AF (2004). Rethinking the emplacement and evolution of zoned plutons: Geochronologic evidence for incremental assembly of the Tuolumne Intrusive Suite. Calif. Geol..

[69] Miller CF, Wark DA (2008). Supervolcanoes and their explosive supereruptions. Elements.

[70] Svensen HH, Polteau S, Cawthorn G, Planke S, Breitkreuz C, Rocchi S (2014). Sub-volcanic Intrusions in the Karoo Basin, South Africa. Physical Geology of Shallow Magmatic Systems.

[71] Ross P-S (2005). Mafic volcaniclastic deposits in flood basalt provinces: A review. J. Volcanol. Geotherm..

[72] Nielsen TFD (2004). The shape and volume of the Skaergaard intrusion, Greenland: Implications for mass balance and bulk composition. J. Petrol..

[73] Annen, C., Latypov, R. M., Chistyakova, S. Yu., Cruden, A. R. & Nielsen, T. F. D. Catastrophic growth of totally molten magma chambers in months to years. *Sci. Adv.* **8**, (in press) (2022). 10.1126/sciadv.abq0394 PMC9506720 36149966

[74] Morse SA, Charlier B, Namur O, Latypov R, Tegner C (2015). Kiglapait intrusion, labrador. Layered Intrusions.

[75] Fourny A, Weis D, Scoates JS (2019). Isotopic and trace element geochemistry of the Kiglapait Intrusion, Labrador: Deciphering the mantle source, crustal contributions and processes preserved in mafic layered intrusions. J. Petrol..

[76] Edmonds M, Cashman KV, Holness M, Jackson M (2019). Architecture and dynamics of magma reservoirs. Philos. Trans. R. Soc. A..

[77] Smith WD, Maier WD (2021). The geotectonic setting, age and mineral deposit inventory of global layered intrusions. Earth Sci. Rev..

