# Supplementary material for: A 5-km-thick reservoir with > 380,000 km3 of magma within the ancient Earth's crust
Source: Sci Rep. 2022 Sep 19;12:15651. doi: 10.1038/s41598-022-19915-w (PMC9485162; doi:10.1038/s41598-022-19915-w)
Supplement: Supplementary file 2 — Supplementary Information 2. [file 41598_2022_19915_MOESM2_ESM.pdf]

Extended data for a paper entitled

# “A 5-km-thick reservoir with >380,000 km$^3$ of magma within the ancient Earth's crust”

by Latypov et al. (2022)

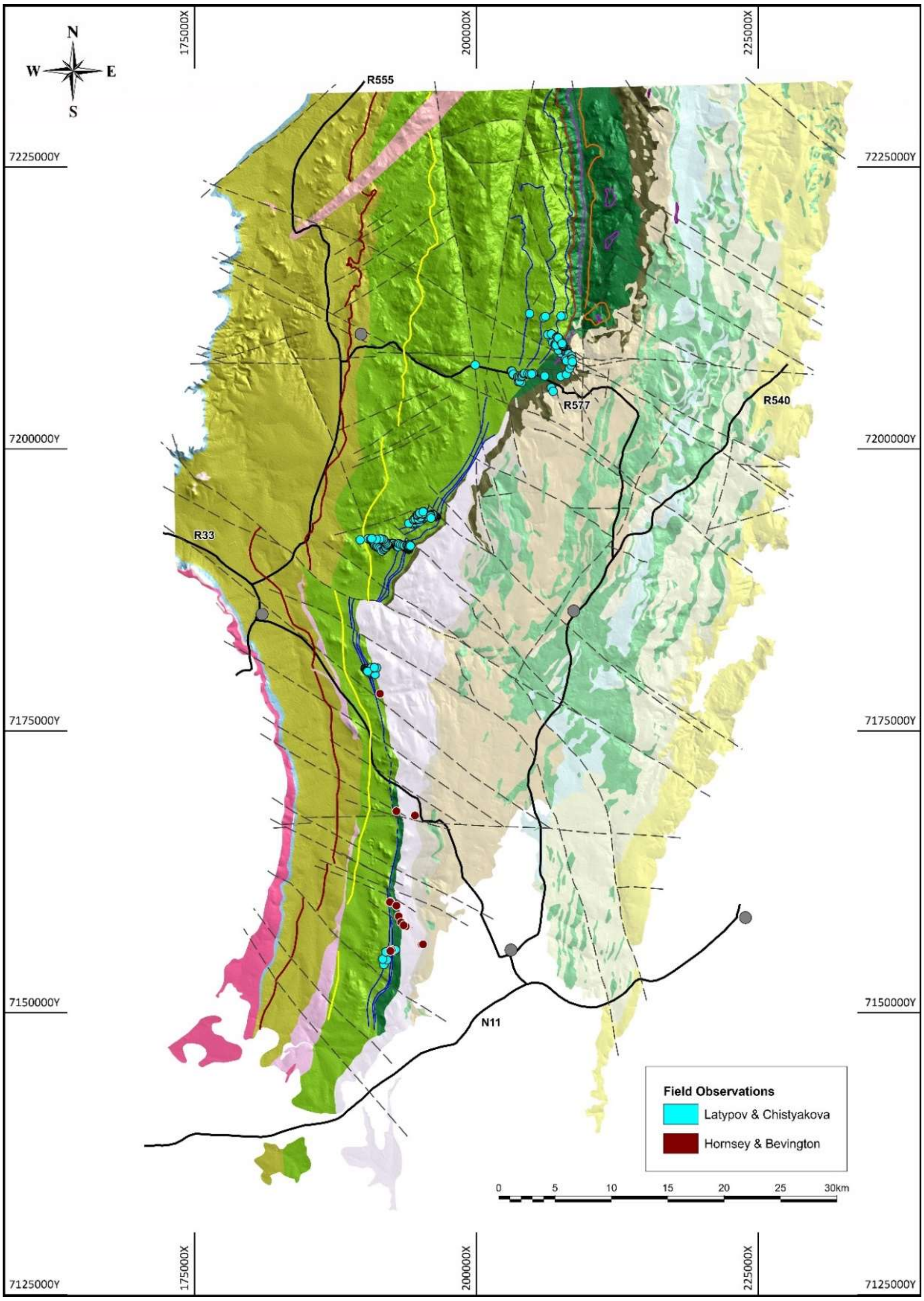


**Extended Data Fig. 1.** Location of GPS points from seven traverses across the Anorthosite Markers at the basal part of the MZ in the southeastern part of the Bushveld Complex. The figure was prepared by Richard Hornsey using Micromine 2021 Release 21.5.

**Thermodynamic modeling**

The crystallization of the Main Zone was modeled using the alphaMELTS 1.9 software[1–4]. The calculations employed the MELTS algorithm, under fractional crystallization condition at 2 kbar, in steps of 5 °C, under FMQ oxygen buffer. The results are synthesized in the Extended Data Fig. 2 and Suppl. Tables SX1 and SX2. There were three steps in the modeling approach:

- step 1 was to identify a melt composition (MZ magma) which by fractional crystallization to produce the zones A, B, C (Fig. 6 and Extended Data Fig. 2);
- step 2 was to identify an opx-saturated melt with a similar sequence of crystallization as MZ-magma, but more primitive (higher Mg# in orthopyroxene and An-content in plagioclase);
- step 3 was designed to model the sequences A' and B' (Extended Data Fig. 2) containing the Pyroxenite Marker, by continuously increase the degree of mixing of an opx-saturated magma obtained at step 2 with a residual magma from stage 1, concomitantly with fractional crystallization of the mix at every calculated step.

Step 1 targeted to identify the parent MZ-magma, that obey the following conditions:

1. The succession of crystallization A-B-C (Extended Data Fig. 2) with the corresponding mineralogy (phases at equilibrium): A = Pl + Cpx + Opx; B=Pl + Cpx + Opx + Pig; C= Pl-Cpx-Pig).
2. The ratio between the calculated volumes to be similar to the ratio between the volumes of cumulates observed in the stratigraphic column.
3. The mineral composition to be similar or close to those observed in the real cumulates, with Mg# in orthopyroxene and An-content in plagioclase continuously decreasing from A to C; the trapped liquid shift effect[5] is to be considered herein, in the shift of both Mg# in orthopyroxene and An-content in plagioclase from higher to lower values.
4. Identifying a residual melt at low temperature with a volume ~ 30% representing the residual-magma (R-magma) that will constitute one endmember of the mix required in step 2.

The starting composition in the calculation of the MZ-magma was identified based on a multiple reiterative calculation (trial-and-error) approach in order to achieve the criteria 1-4 mentioned above. The calculation started from various compositional liquids: tholeiitic MOR-basalt, B2 and B3 compositions of the marginal rocks[6,7], Al-rich basaltic andesites[8]. The preliminary results showed that a typical tholeiitic MORB composition, dry or wet[9] does not produce orthopyroxene at all. Dry MORB produces first plagioclase followed by olivine and then clinopyroxene (Suppl. Table SX1B), whereas the wet MORB gives olivine at liquidus, followed by clinopyroxene and plagioclase (Suppl. Table SX1C). Consequently, MORB cannot be a parent magma for the MZ and this path was abandoned. The B2 composition does not produce orthopyroxene at all (Suppl. Table SX1D). Such melt compositions would produce gabbro cumulates with plagioclase-clinopyroxene and little pigeonite, together with low $Cr_2O_3$ chromite (the latter as liquidus phase). It is clear from the calculations that a composition higher in $SiO_2$ and significantly lower in CaO than B2 (and B3) is needed to reproduce the MZ cumulates. The calculation attempts also showed that a lower Mg# of the starting composition pushes down the appearance of the orthopyroxene very late in the crystallization sequence and decreases the field of orthopyroxene stability to a point where the opx disappears from the assemblage. For example, trying to decrease the Mg# to obtain an opx with low Mg# as observed in the MZ cumulates, the opx disappears from the calculated assemblage leaving its place to pigeonite. This observation supports the interpretation that the

compositions (Mg# in opx) observed today in the cumulates are in fact the result of later equilibration of an earlier-formed opx with a later and colder interstitial liquid (trapped liquid shift effect[5]). This effect is more obvious in the variation of the Mg# in opx than in the An-content in plagioclase. The most challenging aspect of the calculation was to obtain the assemblage opx-cpx-pig-pl at equilibrium, as this equilibrium reflects a very narrow compositional space in the pyroxene world. Once obtained such an equilibrium, a small variation in the starting composition or the temperature fractionation step would break the 4-phase assemblage in such a way that opx or pig would belong to different 3-phase equilibrium assemblages, at different temperatures, and no assemblage contains both opx and pig. It was also noted that increasing $H_2O$ content of the melt promotes the stability of co-existing opx and pigeonite; and increasing the $\log fO_2$ from FMQ to FMQ+2 promotes the stability of magnetite early in the crystallization sequence and extends the stability field of clinopyroxene to higher temperatures (pushing it towards liquidus condition). The result of the modeling, obtained after slightly modifying the starting composition satisfactorily reproduces the above-mentioned criteria (1-4) (see MZ-magma in Table X1, and Extended Data Fig. 2).

The compositions of the identified magmas (MZ, R, and O) are presented in Table X1.

**Table X1:** Compositions of modeled magmas

| Symbol | $SiO_2$ | $TiO_2$ | $Al_2O_3$ | $Fe_2O_3$ | $Cr_2O_3$ | FeO | MnO | MgO | CaO | $Na_2O$ | $K_2O$ | H2O | Mg# |
|---|---|---|---|---|---|---|---|---|---|---|---|---|---|
| MZ-magma | 55.338 | 0.145 | 16.829 | 1.295 | 0.0001 | 6.658 | 0.210 | 5.335 | 8.203 | 4.839 | 0.105 | 1.052 | 58.5 |
| R-magma | 55.844 | 0.164 | 16.481 | 1.384 | 0.0001 | 7.063 | 0.243 | 4.677 | 7.556 | 5.253 | 0.119 | 1.213 | 54.1 |
| O-magma | 55.325 | 0.132 | 17.961 | 0.673 | 0.01 | 3.970 | 0.203 | 8.220 | 10.858 | 2.343 | 0.101 | 0.203 | 78.7 |

The results of running the MZ magma are synthesized in Table X2 (detailed calculations in Suppl. Table SX1A).

**Table X2:** Mineral assemblage, volume, Mg# in orthopyroxene and An-content in plagioclase resulted by fractional crystallization at 2 kbar of MZ-magma

| Assemblage | Cumulate (in Fig X1) | Volume (cm3) | Mg# in opx | An in pl | T (ºC) |
|---|---|---|---|---|---|
| opx-cpx-pl | A | 1.665 | 81.7 | 68 | 1155 |
| opx-cpx-pig-pl | B | 1.527 | 81.2 | 67.5 | 1150 |
| cpx-pig-pl | C | 1.415 | | 67 | 1145 |

Step 2 was to identify by trial-and-error an opx-saturated magma that has a similar sequence of crystallization with the MZ-magma, but it is more primitive (higher Mg# of opx and An-content in plagioclase) in order to reproduce the PM sequence mineralogy (Extended Data Fig. 2, Table X1).

This magma (O-magma) is presented in Table X1. Fractional crystallization of such a magma alone would necessarily produce a continuous decrease in Mg# in opx and An-content in

plagioclase (see Suppl. Table SX2), fact that would not fit the observed variation of these factors in the PM sequence. However, a new influx of O-magma would encounter a resident magma, and we should take into consideration the mixing effect combined with fractional crystallization to reproduce the PM sequence (A' and B').

Step 3 was designed to gradually mix the O-magma (in steps of 10 vol. % of O-magma component) with an R-magma (residual magma from step 1). R-magma was chosen to be the liquid in equilibrium with the cpx-pig-pl in the crystallization sequence of the MZ-magma, as this mineral assemblage represents the cumulates below the PM sequence (C-cumulate in Extended Data Fig. 2). The temperature at which the C-cumulate is in equilibrium with a ~ 30% residual liquid (R-magma) is T=1445 C. With gradually increasing of O-magma in the mix, from $O_{10}R_{90}$ to $O_{70}R_{30}$, coupled with concomitant fractional crystallization in steps of 1 C° the reversals of Mg# and An-content are obtained. The orthopyroxene becomes a liquidus phase at $O_{20}R_{80}$ mix and by increasing the O-magma volume to $O_{30\text{-}40}R_{70\text{-}60}$ ratio the values of Mg# and An-content correspond to the PM appearance (after applying the trapped liquid shift effect[5]). After the formation of PM, if the mixing continues and orthopyroxene is not the only phase to fractionate (vol% of fractionated pl > cpx > opx, see Suppl. Tables S4B-5,6), the Mg# and An-content continue to increase up to a mixing corresponding to $O_{70}R_{30}$. If the O-magma influx and mixing cease at this point, the mixed magma would follow a normal fractional crystallization process and would reproduce the decreasing trends of Mg# and An-content. The results show that gradually changing the ratio of mixing between the residual melt (R-melt) of the MZ-magma with an orthopyroxene-saturated magma (O-magma) reproduced the mineral assemblage and the variation of Mg# in opx and An-content in plagioclase as observed in zones A' and B' (Extended Data Fig. 2, Suppl. Tables 4A and 4B).

The composition of MZ-, R-, and O- magmas are presented in Suppl. Table S1 (extended data), and the MELTS calculations are synthesized in Table S3A and S4A. Detailed calculations are presented in Suppl. Tables S2A, S2B, S2C, S2D, S3B, and S4B, in extended data.

It is intuitive that at different mixing ratios between the two magmas different mineral assemblage and/or modal composition would be produced. For example, a small volume of O-magma (opx-saturated) mixed with a big volume of resident R-magma (plagioclase saturated) would not produce opx as a liquidus phase, as the opx component would be diluted into the bigger volume of the resident magma, which is very depleted in opx and also has a lower Mg# than the O-magma. This means that the base of the PM sequence (A') cannot start with a pyroxenite cumulate.

This exercise of mixing coupled with fractional-crystallization is realized in 9 steps of mixing, from 10% O-magma and 90% R-magma ($O_{10}R_{90}$) up to $O_{90}R_{10}$ and the results are presented in Suppl. Table 4a and 4B (see individual tabs for each step of the calculation), summarized in Suppl. Table S5 and Extended Data Fig. 2.

The orthopyroxene that starts to crystalize at the beginning of such mix (following the crystallization of plagioclase and cpx) would have a lower Mg# than the opx that would have

crystallized from the O-magma alone, and it would have an Mg# higher than the opx from the resident melt, producing the start of the reversal observed in Extended Data Fig. 2, below the PM. It is also intuitive that by the continuous increase of the volume of opx-saturated O-magma into the mix, the solids fractionated from the mix at each calculated step will change the modal composition of the cumulates, producing a gradual increase of opx up to the point where the orthopyroxene becomes a liquidus phase and can fractionate a pyroxenite (such as the PM). At the same time with increasing volume % of the opx, the Mg# in opx and An-content in plagioclase will gradually increase, proportionally to the increasing volume of the opx-saturated and more primitive O-magma into the mix. Observe in Extended Data Fig. 2 that the inflections of the modeled Mg# in opx and An-content in plagioclase match the observed ones in the PM sequence stratigraphy (the liquid-sift effect is to be considered) and that opx becomes a liquidus phase starting with $O_{20}R_{80}$ mix, only. It produces the PM at $O3_{0-40}R_{70-60}$ ratio and the mixing stopped at ~ $O_{70}R_{30}$ ratio. It is obvious from this exercise that the PM sequence does not start with a pyroxenitic layer but rather a mela-noritic cumulate below PM that continuously grades into a pyroxenitic composition, with gradually increasing Mg# in opx and An-content in plagioclase up to the point when the O-magma becomes dominant (~$O_{70}R_{30}$) or when the R-magma is exhausted (completely mixed). At this point, the crystallization reaches the maximum of Mg# in opx and An-content in plagioclase (the maximum of the Mg# and An-content reversals in Extended Data Fig. 2). The crystallization that follows the complete mixing would produce a decrease of opx vol% in the cumulate, as well as the expected decrease of Mg# in opx and An-content in plagioclase. This exercise elegantly explains the fact that the new influx of the opx-saturated magma into the upper part of the MZ (Zone A') does not start with the PM marker but starts below it, where the reversal of Mg# in opx and An-content in plagioclase starts.

The gradual reversal of Mg# in opx and An-content in plag below the PM is easily explained by mixing coupled with fractional crystallization of a magma that resulted by a gradual increase of mixing between a new influx of opx-saturated magma (O) into an existent volume (ca. 30 vol % in respect to the existing C-cumulates before the new influx of magma) of a plagioclase-saturated resident (R) magma.

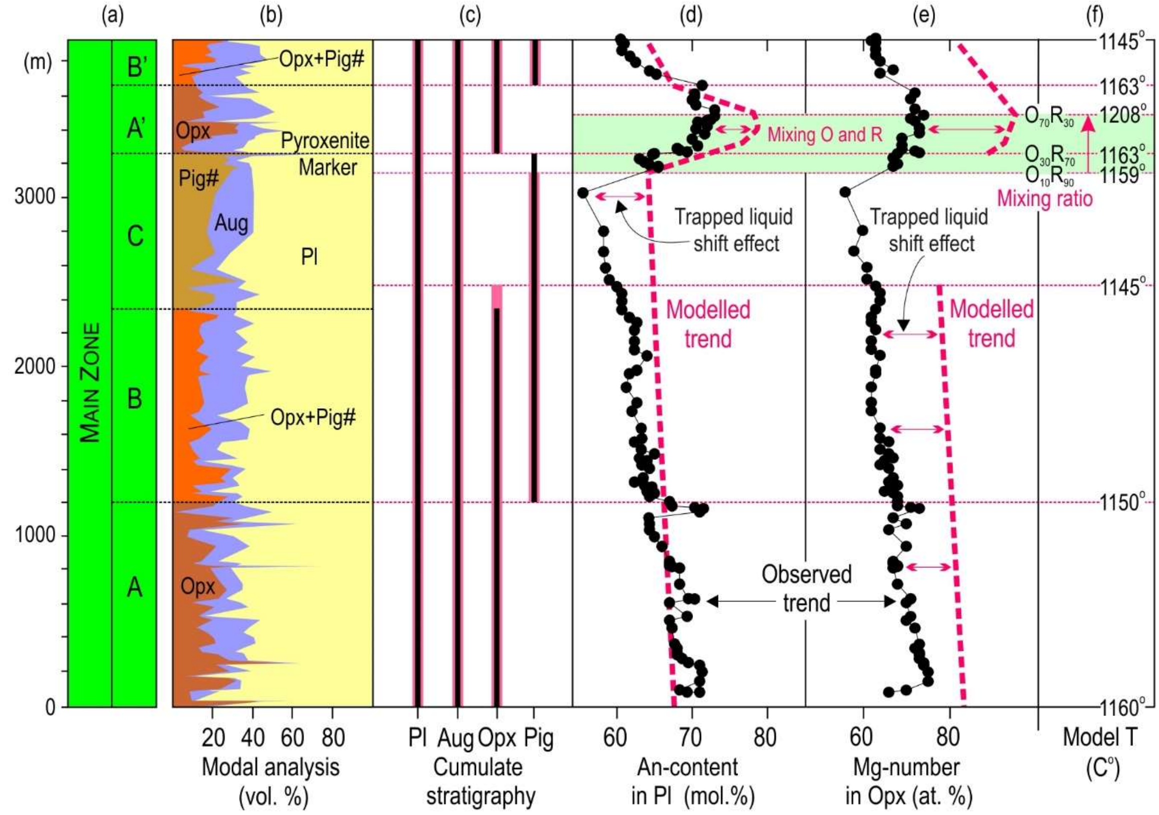


**Extended Data Fig. 2. Modal abundances, cumulate stratigraphy and compositional variations of minerals through the Main Zone in the Tonteldoos area of the southeastern part of the Bushveld Complex.** The section is approximately along the transect line CD in Figs. 1 and 2. The lower stratigraphy (a-c) is characterized by a systematic crystallization sequence Opx+Aug+Pl (A), Opx+Aug+Pig+Pl (B), and Aug+Pig+Pl (C). The uppermost stratigraphy (a-c) is marked by re-appearance of cumulus Opx at the Pyroxenite Marker so that the crystallization sequence Opx+Aug+Pl (A') and Opx+Aug+Pig+Pl (B') is repeated. The lower stratigraphy (d-e) shows systematic evolutionary trends in composition of plagioclase (decrease in An-content, 100Ca/(Ca+Na)) and orthopyroxene (decrease in Mg-number, 100Mg/(Mg + Fe)). The transition to the Pyroxenite Marker (d-e) is characterized by a gradual compositional reversal towards higher An-content of plagioclase and Mg-number of orthopyroxene. All compositional data in (a-e) are from Gruenewaldt[10] and are summarized in Supplementary Data Table 1. The trapped liquid shift[5] in composition of Mg-number in orthopyroxene and An-content in plagioclase represent the re-equilibration of the minerals with the trapped liquid during cooling at the post-cumulate stage. The modelling results are explained in the electronic appendix (Section "Thermodynamic modelling"), summarized in SuplTables S3A, S4a and S5, and presented in detail in SuplTables S3B and S4B-1 to S4B-7). O, orthopyroxene-saturated magma; R, resident melt; Pl, plagioclase; Opx, orthopyroxene; Aug, augite; Pig#, inverted pigeonite. The figure is prepared by Sofya Chistyakova and Gelu Costin using CorelDRAW (version 18.1.0.690).

**References:**


1. Ghiorso, M. S., Hirschmann, M. M., Reiners, P. W. & Kress, V. C. The pMELTS: A revision of MELTS for improved calculation of phase relations and major element partitioning related to partial melting of the mantle to 3 GPa. *Geochem. Geophys. Geosystems.* **3**, 1030 (2002).
2. Smith, P. M. & Asimow, P. D. Adiabat_1ph: A new public front-end to the MELTS, pMELTS, and pHMELTS models. *Geochem. Geophys. Geosyst.* **6**, 1–8 (2005).
3. Asimow, P. D., Dixon, J. E. & Langmuir, C. H. A hydrous melting and fractionation model for mid-ocean ridge basalts: Application to the Mid-Atlantic Ridge near the Azores. *Geochem. Geophys. Geosyst.* **5**, Q01E16 (2004).
4. Ghiorso, M. & Sack, R. Chemical mass transfer in magmatic processes IV. A revised and internally consistent thermodynamic model for the interpolation and extrapolation of liquid-solid equilibria in magmatic systems at elevated temperatures and pressures. *Contrib. Mineral. Petrol.* **119**, 197–212 (1995).
5. Barnes, S. J. The effect of trapped liquid crystallization on cumulus mineral compositions in layered intrusions. *Contr. Mineral. and Petrol.* **93**, 524–531 (1986).
6. Barnes, S.-J., Maier, W. D. & Curl, E. A. Composition of the marginal rocks and sills of the Rustenburg Layered Suite, Bushveld Complex, South Africa: Implications for the formation of the platinum-group element deposits. *Econ. Geol.* **105**, 1491–1511 (2010).
7. Yang, S.-H. *et al.* Parental magma composition of the Main Zone of the Bushveld Complex: evidence from in situ LA-ICP-MS trace element analysis of silicate minerals in the cumulate rocks. *J. Petrol.* **60**, 359–392 (2019).
8. Latypov, R. *et al.* Platinum-bearing chromite layers are caused by pressure reduction during magma ascent. *Nat. Commun.* **9**, (2018).

9. Allan, J. F., Batiza, R., Perfit, M. R., Fornari, D. J. & Sack, R. O. Petrology of lavas from the Lamont Seamount Chain and adjacent East Pacific Rise, 10° N. *J. Petrol.* **30**, 1245–1298 (1989).

10. Von Gruenewaldt, G. The Main and Upper zones of the Bushveld Complex in the Roossenekal area, eastern Transvaal. *Trans. geol. Soc. S. Afr.* **76**, 53–61 (1973).

**Suppl. Table captions**

Table SX1A: The alphaMELTS results for fractional crystallization in steps of 5  C of MZ-magma at 2 kbar

Table SX1B: The alphaMELTS results for fractional crystallization in steps of 5  C of dry MORB magma at 2 kbar (MORB data after Allan et al., 1989)

Table SX1C: The alphaMELTS results for fractional crystallization in steps of 5  C of wet MORB magma at 2 kbar (MORB data after Allan et al., 1989)

Table SX1D: The alphaMELTS results for fractional crystallization in steps of 5  C of B2 magma at 2 kbar (B2 composition after Barnes et al., 2010)

Suppl. Table SX2: Results of alphaMELTS calculations for fractional crystallization of O-magma (opx-saturated) at 2 kbar

Suppl. Table SX3-1: Mixing O-and R-magmas in steps of 10 vol% of O-magma concomitantly with fractional crystallization at 2 kbar: O=10 vol%, R=90 vol %

Suppl. Table SX3-2: Mixing O-and R-magmas in steps of 10 vol% of O-magma concomitantly with fractional crystallization at 2 kbar: O=20 vol%, R=80 vol %

Suppl. Table SX3-3: Mixing O-and R-magmas in steps of 10 vol% of O-magma concomitantly with fractional crystallization at 2 kbar: O=30 vol%, R=70 vol %

Suppl. Table SX3-4: Mixing O-and R-magmas in steps of 10 vol% of O-magma concomitantly with fractional crystallization at 2 kbar: O=40 vol%, R=60 vol %

Suppl. Table SX3-5: Mixing O-and R-magmas in steps of 10 vol% of O-magma concomitantly with fractional crystallization at 2 kbar: O=50 vol%, R=50 vol %

Suppl. Table SX3-6: Mixing O-and R-magmas in steps of 10 vol% of O-magma concomitantly with fractional crystallization at 2 kbar: O=60 vol%, R=40 vol %

Suppl. Table SX3-7: Mixing O-and R-magmas in steps of 10 vol% of O-magma concomitantly with fractional crystallization at 2 kbar: O=70 vol%, R=30 vol %

Suppl. Table SX3-8: Mixing O-and R-magmas in steps of 10 vol% of O-magma concomitantly with fractional crystallization at 2 kbar: O=80 vol%, R=20 vol %

Suppl. Table SX3-9: Mixing O-and R-magmas in steps of 10 vol% of O-magma concomitantly with fractional crystallization at 2 kbar: O=90 vol%, R=10 vol %
